# *Dscam2* affects visual perception in *Drosophila melanogaster*

**DOI:** 10.3389/fnbeh.2015.00149

**Published:** 2015-06-09

**Authors:** Danny S. Bosch, Bruno van Swinderen, S. Sean Millard

**Affiliations:** ^1^School of Biomedical Sciences, The University of QueenslandSt. Lucia, QLD, Australia; ^2^Queensland Brain Institute, The University of QueenslandSt. Lucia, QLD, Australia

**Keywords:** Dscam, *Drosophila*, visual perception, behavioral assay, motion detection

## Abstract

Dscam2, a cell surface protein that mediates cellular repulsion, plays a crucial role in the development of the *Drosophila melanogaster* visual system. Dscam2 generates boundaries between neighboring modules in the fly optic lobe; in *Dscam2* mutants this visual system modularity is compromised. Although developmental wiring defects have been well described in the *Dscam2* mutant, behavioral consequences have not been investigated. To address this, we examined the visual behavior of *Dscam2* mutant flies. Using a phototaxis assay, we ascertained that these flies are not blind, but have a reduced phototaxic response. Through population-based and single fly optomotor assays, we found that *Dscam2* mutant flies can track motion but that their response is opposite to control flies under defined experimental conditions. In a fixation paradigm, which allows tethered flies to control the angular position of a visual stimulus, mutant flies' responses were diametrically opposed to those seen in control flies. These data suggest that modest changes in the modularity of the fly visual system in the *Dscam2* mutant can dramatically change the perception of specific visual cues and modify behavior.

## Introduction

The brain is the most complex organ in the human body as it utilizes an organized network of billions of neurons. These neurons relay sensory information into actions and are the foundation of unique behaviors. In order to establish a functional connective network, neurons need to be able to discriminate and identify not only their own neurites but also those of their neighboring cells. This discrimination is typically achieved through cell recognition molecules (CRMs) expressed on the plasma membrane. Protein-protein interactions between different neurons play crucial roles in generating distinct boundaries in the brain, in guiding neurons to their targets, and in promoting synaptogenesis between pre- and postsynaptic cells (Tessier-Lavigne and Goodman, 1996).

**Figure 1:**
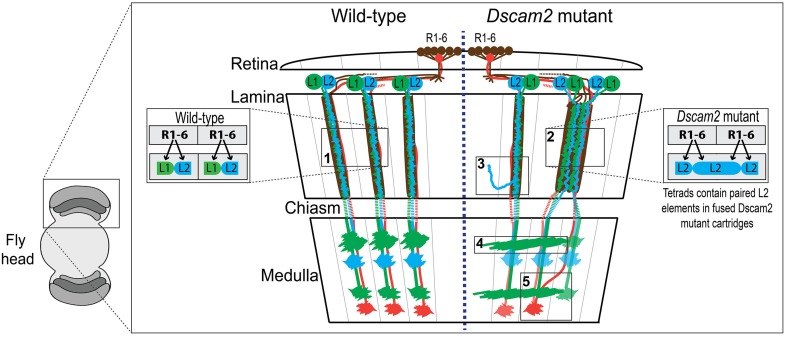
**Wiring diagram of the optic lobes of wild type and the** ***Dscam2*** **mutant**. Schematic of wild type (WT) (left-side) and *Dscam2* mutant (right-side) optic lobes with wiring details highlighted in boxes. Photoreceptors R1-6 (brown) in the retina form synapses with a postsynaptic complex that includes L1 (green) and L2 (blue) lamina neurons. Photoreceptors R7 (red) and R8 (not-shown) project directly to the medulla. (1) In WT animals, L1 and L2 are postsynaptic at every photoreceptor synapse. (2) In *Dscam2* mutant animals, 40% of the photoreceptor synapses within fused cartridges contain two L2 elements, one of which is from a neighboring cartridge. (3) L2 dendritic arbors project into neighboring lamina cartridges when they lack *Dscam2*. (4) *Dscam2* restricts L1 lamina neuron axons to a single column in the medulla. (5) R7 and R8 axons are disorganized in *Dscam2* mutant animals frequently crossing over into adjacent medulla columns; this phenotype is non-autonomous.

Studying how neurons establish connections in the mammalian brain is challenging due to the sheer complexity of information carried within the central nervous system. The *Drosophila melanogaster* brain represents a simpler system that abides by similar rules in terms of neural connectivity and development (Meinertzhagen and Hanson, 1993; Clandinin and Zipursky, 2002). In the retina, photoreceptors (R-cells) are organized into ~750 modular units called ommatidia (Figure 1). Each ommatidium contains one of each type of photoreceptor (R1-8). The motion-detecting R1-6 cells extend axons to the lamina, where they form ~750 synaptic units called lamina cartridges. Within each cartridge R1-6 axons form a cage around two postsynaptic cells, lamina neurons L1 and L2. Each photoreceptor terminal makes ~300 output synapses, each containing four postsynaptic elements two of which are invariantly lamina neurons L1 and L2 (Figure 1, inset box 1). L1 and L2 axons project to the medulla where they form connections within a single medulla module called a column. Motion detection relies on the modularity of the visual system as it compares the timing of activation between modules, as suggested in the Hassenstein-Reinhardt Elementary Motion Detection (EMD) model (Hassenstein and Reichardt, 1956; Borst and Egelhaaf, 1989). Therefore, disruption of boundaries between cartridges and columns would be expected to affect motion tracking in the fly.

Down Syndrome Cell Adhesion Molecule 2 (Dscam2), a homolog of human DSCAM, is a cell recognition molecule crucial for the modular development of the *Drosophila* visual system (Millard et al., 2007). Dscam proteins in all species mediate homophilic binding, but Dscam2-Dscam2 interactions in the fly have been shown to induce repulsion, at least in the neurons studied thus far (Millard et al., 2007). During development, Dscam2 generates boundaries between neighboring modules in the visual system through the process of cell-type-specific homophilic repulsion. For example, Dscam2 restricts L1 lamina neuron axons to a single column in the medulla (Millard et al., 2007). When *Dscam2* is removed from these cells, they make inappropriate connections in adjacent columns (Figure 1, box 4). L2 dendrites exhibit a similar phenotype when they lack *Dscam2*, as mutant dendritic arbors project into adjacent lamina cartridges (Lah et al., 2014) (Figure 1, box 3). Given that identical synaptic targets for L1 axons and L2 dendrites exist in adjacent columns and cartridges, respectively, these mutant cells are likely forming synapses in neighboring regions of the optic lobe, thereby reducing the number of independent modules. Direct evidence for this has been observed at photoreceptor synapses of *Dscam2* homozygous mutant animals (Millard et al., 2010). In the lamina of the *Dscam2* mutant, cartridges frequently fuse. L2 dendrites are postsynaptic to R cells from both cartridges within the fused cartridge, something that never happens in wild-type animals. Approximately 40% of the synapses within these fused cartridges contain two L2 dendrites and no L1 (Figure 1, box 2). Thus, a visual stimulus that would normally engage one of the cartridges, engages both in the mutant. Lastly, R7 and R8 axons are disorganized in *Dscam2* mutant animals. R7 and R8 frequently cross over into adjacent medulla columns; this non-autonomous phenotype (Millard et al., 2007) also results in a reduction of modularity (Figure 1, box 5). Together, these morphological and synaptic phenotypes in the optic lobe suggest that Dscam2 plays a major role in establishing modularity.

The changes in modularity in the optic lobes of *Dscam2* mutants provide an ideal opportunity to investigate how this iterated structure contributes to visual processing. Visual behaviors have been studied extensively in *Drosophila* (Paulk et al., 2013). Simple phototaxic behaviors, which measure a fly's propensity to move toward light (Benzer, 1967) have been developed as have more complex motion tracking assays, in which flies fly or walk in the direction of a moving grating (Blondeau and Heisenberg, 1982; Zhu and Frye, 2009). Assays that measure how a fly orients itself in relation to an object (pattern induced visual-orientation) have been used to study more complex behaviors (Reichardt and Poggio, 1976; Heisenberg and Wolf, 1979; Wolf and Heisenberg, 1980).

In this study we investigated whether the lack of *Dscam2* altered visual perception in *Drosophila*. To examine the flies' visual perception, we tested their phototaxic, motion tracking, and pattern induced visual-orientation capabilities. We found that *Dscam2* mutants have defects both in detecting light and motion. However, these phenotypes are conditional: under certain experimental conditions the mutants perform as well as controls. Interestingly, in three different visual behavior paradigms we found that *Dscam2* mutants exhibit a behavioral response that is opposite to control flies when exposed to specific visual stimuli. We conclude that the disrupted modularity of the *Dscam2* mutant visual system lowers their visual acuity and inverts their behavioral response to specific stimuli.

## Materials and methods

### *Drosophila* stocks

*Dscam2* mutant stocks (*Dscam2*^*null*−1^, *Dscam2*^*null*−2^, and *Dscam2*^*null*−3^, previously generated by homologous recombination, (Millard et al., 2007) were isogenized in a *w*^1118^ background through eight backcrosses. After isogenization the *w*^1118^ X chromosome was replaced with a *w*^+^. The final genotype contained an X chromosome from *Canton-S* (sourced from the van Swinderen laboratory, Queensland Brain Institute, The University of Queensland, Australia) and a second chromosome from *w*^1118^ background. The third chromosome was either from the *w*^1118^ background, for the control flies, or a recombinant *w*^1118^ third chromosome that contained the *Dscam2*^*null*^ mutant allele. Flies were reared on standard *Drosophila* yeast-based media and kept at 22 − 25°C under 12-h light and 12-h dark cycles.

### Population visual response assay

#### Fly preparation

Female flies were collected (*n* = 27−33) between 4 and 12 days after eclosion by CO_2_ anesthesia, the day before the experiment. Flies were starved at room temperature for 19–22 h in modified disposable polyethylene “jumbo” transfer pipettes (Thermo Fisher Scientific, Waltham, Massachusetts) containing 10 μl of water prior to experimentation. All the experiments were conducted between early and mid-afternoon to reduce variation between different groups.

#### Phototaxis

**Figure 2:**
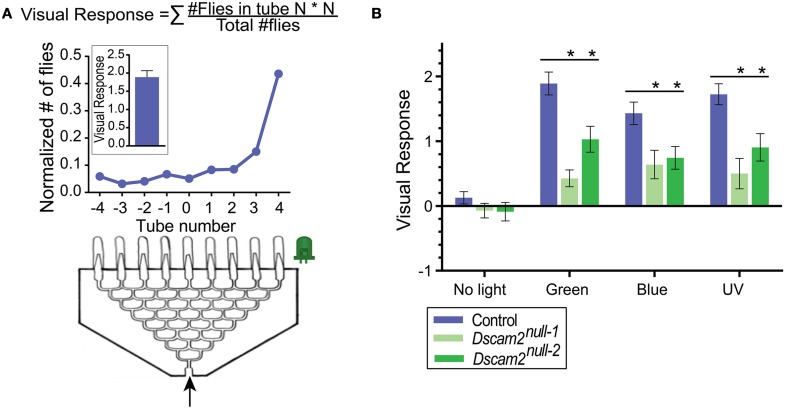
***Dscam2*** **mutants have a reduced phototaxic response in the startle-induced phototaxis assay. (A)** Phototaxis assay. Flies (~30 per maze) enter the maze and end up in one of the nine end tubes (−4 to 4). The normalized distribution of flies from one maze is shown in the line graph. This was calculated by dividing the number of flies in each tube by the total. The visual response formula is at the top of the panel and the response of the maze shown is plotted in the inset. **(B)** Phototaxis of control (blue), *Dscam2*^*null*−1^ mutant (light green) and *Dscam2*^*null*−2^ mutant (green) to different light sources. The mutant response is impaired compared to control. Student's *t*-test was performed for normal distributed data and Mann-Whitney test for non-normal distributed data, ^*^p < 0.05. For all groups at least nine mazes of approximately 30 flies each were run for every condition. Error bars indicate SEM.

For phototaxis, an eight-point choice maze (J&M Specialty Parts, San Diego, California, USA) was used as described previously in van Swinderen and Flores (2007) (Figure 2A). The maze has eight tiers and the flies make a choice to turn left or right at each tier. At the end of the maze, flies are collected at one of nine different exit points. A UV (360–363 nm), green (528 nm) or blue (472 nm) LED light (NS360L-3RIQ, B3B-443-B525, B3B-447-1x Rothner Lasertechnik, Vienna, Austria), powered by a standard 9-volt battery, was used as the light source and placed to the left or the right of the maze exit points. The tube containing the flies was tapped once to startle the flies and then inserted into the maze entrance. On average, it took a group of 30 flies 2.5 min to reach one of the nine exit points of the maze. Here, they were automatically counted using infrared sensors (modified *Drosophila* Activity Monitors, Trikinetics, Waltham, Massachusetts) (Evans et al., 2011). A single experiment consisted of one run with the light source on the left and the second run with a new group of flies and the light source on the right side. At least five experiments with 10 mazes and approximately 30 flies each were performed per stimuli, resulting in at least ~300 flies per data point. The light source was removed for the negative control. A visual response was calculated from fly counts with custom-written Matlab (version R2013b, MathWorks, Natick, MA) scripts as a weighted average of the number of flies in each exit point of the maze (Visual response = (# flies in tube N)^*^N/(total # of flies), where N = tube number −4 to +4) (van Swinderen and Flores, 2007) (Figure 2A).

#### Moving visual stimuli

Experiments were performed using a similar method described above for the phototaxis experiments, except with moving visual stimuli. The behavioral arena was similar to that described by van Swinderen and Flores (2007), except that LED panels were used instead of CRT monitors due to their higher refresh rate (>160 Hz), which is above the flicker fusion frequency for *Drosophila* vision (Heisenberg, 1984). A diagram of the set-up is presented in Figure S1.

#### Visual stimuli

Visual stimuli were programmed in VisionEgg (Straw, 2008) using Python programming language. One experiment consisted of the visual stimulus presented right-to-left to one group of flies and left-to-right to a different group of flies to prevent bias. At least four experiments with eight mazes with approximately 30 flies each were performed per visual stimulus, thus resulting in at least ~240 flies per data point. The standard grating stimulus was a moving grating of black and green bars with a contrast of 1, a spatial frequency of 0.018 cycles/°, a temporal frequency of 3 cycles/s and a velocity of 164.4°/s. The contrasts used were defined as the difference between light (LED on) and dark (LED off) values [intensity (I) max, I min, respectively] divided by the sum of light and dark values. The standard condition with a contrast of 1 had an I max of 594 lx and an I min of 0 lx. The contrast, spatial frequency and temporal frequency were adjusted using VisionEgg software. We tested a range of contrast levels (0.1–1, e.g., low to high level of contrast), temporal frequency levels (1–16 cycles/s), and spatial frequency levels (0.0095 to 0.0402 cycles/°). The velocity of the grating was kept constant at of 164.4°/s for the different spatial frequency levels.

#### Data analysis and statistics

Data analysis was performed with custom-written Matlab scripts and statistical analysis by use of Graphpad Prism 5.00 (Graphpad software, San Diego California USA). For all stimuli an average and standard error of the mean (SEM) visual response was calculated, based on individual visual response experiments performed over multiple days. A Lilliefors test (Lilliefors, 1967) was used to test for normality of the dataset. To compare the average visual response of the mutant with that of control, we performed the student *t*-test for normally distributed data and a Mann-Whitney for non-normally distributed data. When multiple groups were compared we performed a two-way ANOVA. Group size varied from n = 8 − 62 per group and per experiment. Statistics for the maze results are shown in Supplementary Table 1.

### Single fly optomotor and fixation assay

#### Fly preparation

Female adult flies were collected between 1 and 6 days after eclosion by use of CO_2_ anesthesia, 2 to 4 days before the experiment and placed in a vial with fresh food. In contrast with the visual response maze assay, flies were not starved but kept on fresh food at room temperature. All the experiments were performed between early and mid-afternoon to reduce variation.

#### Tethered-walking set-up

For optomotor and fixation experiments, 3 to 10-day-old female flies were cold-anesthetized. The head, thorax and wings where tethered to a 0.1 mm thick tungsten rod with blue light activated dental cement (Whaledent AG, Switzerland). After 2 to 5 h recovery, the animals were placed in the tethered-walking set-up consisting of a diamond-shaped arena of four LED panels. Each panel contained one blue, green and red LED in a 32 × 32 pixel configuration. This set-up is similar to that used in Paulk et al. (2014), except that ball movement was monitored by a camera (Point Grey, Richmond BC, Canada), rather than infra-red sensors (Moore et al., 2014).

#### Optomotor experiments

Similar to the visual response maze, in the optomotor assay a moving grating composed of green and black bars was used as the visual stimulus. The standard grating stimulus had a contrast of 0.6, a spatial frequency of 0.051 cycles/°, temporal frequency 3 cycles/s and a velocity of 58.8°/s. The contrast, spatial frequency and temporal frequency could be adjusted with VisionEgg software. The contrasts used were defined as the difference between light (LED on) and dark (LED off) values (I max, I min, respectively) divided by the sum of light and dark values. The standard condition with a contrast of 1 had an I max of 594 lx and an I min of 0 lx. We tested a range of different temporal frequency levels (9.8–104.4 cycles/s) and spatial frequency levels (0.026–0.102 cycles/°). The velocity of the grating was kept constant at 58.8 or 14.7°/s for the different spatial frequency levels. The spatial frequencies were calculated based on the distance from the front of the fly to the middle of the two front panels (13 cm). Due to the configuration of the arena, the distance from the fly to the middle of the two back panels was 8 cm. This leads to a difference in spatial wavelength between front and back panels. For example, a visual stimulus with a spatial frequency of 0.051 cycles/° would have a spatial wavelength of 19.6° in the front and 33.4° in the back of the arena.

#### Fixation experiments

Fixation behavior was measured using a closed-loop visual stimulus in which the fly is able to control the position of the 6-pixel wide and 32 pixel high bar. Computer-driven random displacement of the bar was used to ascertain fixation behavior. Following bar displacement, the fly rotates the ball to reposition the bar. Two different stimuli were used: (1) a fixation stimulus (dark bar on a light background) of which control flies on average place the bar to the front 180° of the arena and (2) an anti-fixation stimulus (light bar on a dark background) which on average control flies place directly behind them in the arena.

#### Visual stimuli

Visual stimuli were programmed in VisionEgg (Straw, 2008) using Python programming language. For moving grating experiments, one experiment consisted of a clockwise optomotor stimulus presented for 90 s followed by 60 s of a green screen and then by 90 s of counter-clockwise stimulus. For fixation experiments, one experiment consisted of 3 min presentation of a fixation or anti-fixation stimulus. This stimulus was randomly displaced 84° clockwise or counter-clockwise every 5 to 18 s to keep the flies engaged with the stimuli. Flies were exposed to seven to 12 displacements per experiment.

#### FicTrac

To monitor fly locomotion on the air-supported ball, we used FicTrac, which tracks spherical motion of the moving ball. From these data points a two-dimensional fictive path can be generated (Moore et al., 2014). This information can be used to graph turning of the ball over time, and an average angular velocity (the response to the optomotor stimulus).

#### Data analysis and statistics

Significance between control and mutant flies was calculated as described in data analysis and statistics for the visual response maze. Group size for optomotor experiments varied from n = 8–32 per group and per visual stimuli condition. For fixation experiments, the location of the bar (in pixels) at each sampling point was recorded and transformed to a 360° location around the fly. From these data, a radial histogram was plotted by using the circular statistical toolbox in Matlab (Berens, 2009). Using the same toolbox, a mean vector angle and the shape of the distribution (resultant vector length) was determined per fly. From these individual mean directions and resultant vector lengths, a group mean direction and resultant vector length was determined for each genotype. The group mean direction is graphically represented in the radial histogram as an arrowhead. We tested for non-uniformity of the circular data using a Rayleigh test on the group mean directions. Significance (*p* < 0.05) is indicated by a black asterisk. Resultant vector lengths from individual flies of a genotype are graphically represented in box plots with min to max whiskers in which the color of the median line corresponds with the color of the arrowhead (indicating direction) in the radial histogram. The mean direction was compared to a specified mean direction of 0 or 180° using a one-sample test for the mean angle. The mean direction and resultant vector lengths between genotypes were compared using a Watson-Williams multi-sample test to test for equal mean directions and a Kruskal-Wallis test for equal vector lengths. These circular statistics are shown in Supplementary Tables 2–4. Group size varied from n = 8–21 per visual stimuli condition.

## Results

### *Dscam2* mutant flies have a reduced phototaxic response

Many insects including *Drosophila* exhibit positive phototaxic behavior, in which they are attracted to a light source (Benzer, 1967). To determine whether *Dscam2* mutant flies detect light, we set up a phototaxis assay using an eight-point choice maze. In this assay, a group of flies respond to a light source at one end of the maze (Figure 2A). We tested phototaxis toward green, blue and UV light and calculated a visual response based on the distribution of flies at the end of the maze (see Materials and Methods). Control flies (see Materials and Methods) responded strongly to all three wavelengths by walking toward the light, resulting in a skewed distribution of flies toward the light source at the end of the maze (Figure 2B). Approximately 60% of the flies ended up in the three exit tubes closest to each light source, and these responses were not significantly different from each other (student *t*-test, *p* > 0.05). In control experiments where no light was present, flies distributed evenly along the exit tubes, resulting in a visual response close to zero. Two different strains of *Dscam2* mutant flies displayed a significant response to all three light sources with between 30 and 45% of the flies choosing the three exit tubes closest to the light source (Figure 2B). The responses of the *Dscam2* mutant flies to the three wavelengths were not significantly different from each other (student *t*-test, *p* > 0.05) and no response was evoked without light (Figure 2B). For all three wavelengths, the magnitude of the response in the mutants was significantly reduced (student *t*-test, *p* < 0.05) compared to controls (Figure 2B). These results suggest that the circuitry that controls light detection is functional, but impaired, in the *Dscam2* mutants.

### *Dscam2* mutant flies turn against the direction of motion in a population assay

**Figure 3:**
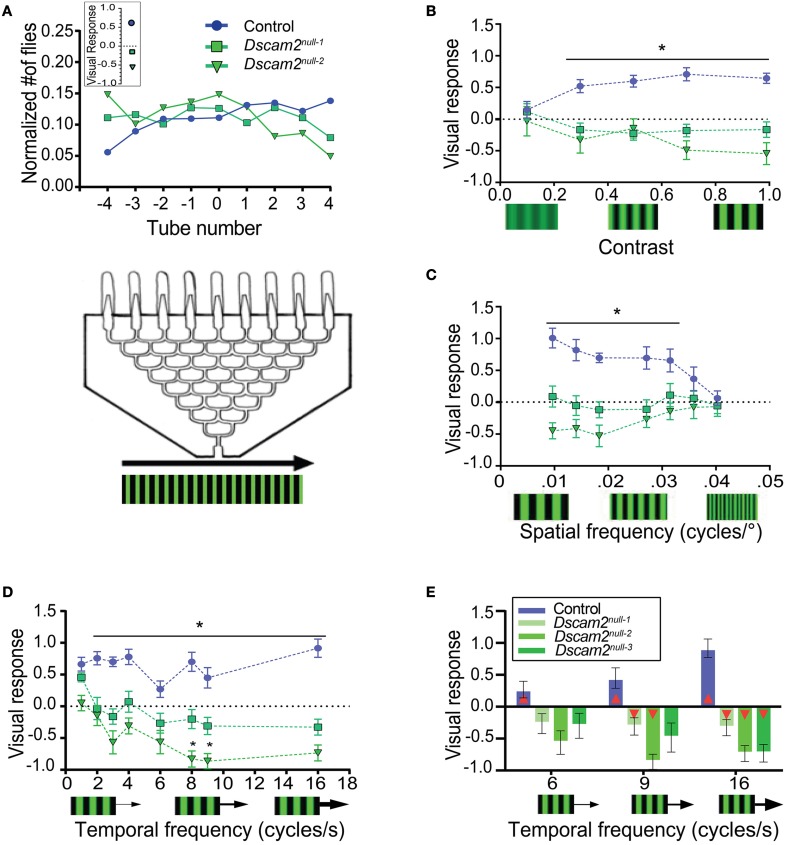
***Dscam2*** **mutants have a weakened and inverted response to motion in the population visual response assay. (A)** Population visual response assay. The same maze that was used for the phototaxis assay was placed on LED panels displaying moving gratings. The line graph displays the normalized distribution of flies in the maze. On average, control flies follow the direction of motion, resulting in a positive visual response. *Dscam2* mutants exhibit the opposite behavior, resulting in a negative response. The inset represents the calculated visual response for each genotype (see Materials and Methods). **(B–D)** Visual responses of control, *Dscam2*^*null*−1^ and *Dscam2*^*null*−2^ to different grating parameters. **(B)** Visual responses to different grating contrasts; **(C)** spatial frequencies and **(D)** temporal frequencies. ^*^p < 0.05 (control *vs* mutants). **(E)** A third *Dscam2* mutant (*Dscam2*^*null*−3^, dark green bar) behaves most similarly to *Dscam2*^*null*−1^ over different temporal frequencies. Red triangles within bars indicate a significant difference from zero. For all groups at least eight mazes of approximately 30 flies each were run for every condition. Error bars indicate SEM.

To assess the ability of flies to track motion, we placed the maze described above on top of LED panels displaying moving visual stimuli. A visual response was calculated based on the distribution of flies at the maze exits (see Materials and Methods). The visual response was defined as positive when flies followed the direction of motion and negative when they moved against the direction of motion. When control flies were exposed to a standard grating of black and green stripes with a contrast level of 1 (see Materials and Methods), a spatial frequency of 0.051 cycles/degree and a temporal frequency of 3 Hz, they followed the direction of the motion, resulting in a visual response of 0.64 ± 0.1 (Figure 3A). This response was similar to previously published results using wild-type flies (Evans et al., 2011). *Dscam2* mutant flies, however, moved against the direction of the motion with this stimulus, resulting in a negative visual response of −0.16 ± 0.12 and −0.54 ± 0.17 for *Dscam2*^*null*−1^ and *Dscam2*^*null*−2^, respectively (Figure 3A). Control and mutant flies followed rightward and leftward motion with similar magnitudes, as expected.

To further investigate how the absence of *Dscam2* affects visual behaviors, we challenged the visual system of the flies by altering the contrast, spatial frequency and temporal frequency of the moving grating. Each of these parameters contributes to how well an image is detected and therefore provides a means for testing the sensitivity and acuity of the visual system. In general, the mutant flies perceived changes in these visual parameters, but they consistently responded by turning against the direction of motion.

Control flies had a contrast-dependent behavioral response to moving gratings, with no response to a low contrast stimulus (0.1) and a maximal response at higher contrasts (0.7) (Figure 3B). *Dscam2* mutant flies also responded to changes in contrast, but the magnitude of the response was different between the two mutants. *Dscam2*^*null*−1^ flies elicited weaker responses than the *Dscam2*^*null*−2^ flies. Interestingly, both mutants moved against the moving grating, rather than following the direction of motion like the controls (Figure 3B). Similar results were obtained when the spatial frequency (the width of the bar) was varied. Control flies responded well to low spatial frequencies (wider bars) but this response reduced to zero at higher spatial frequencies (narrower bars). The responses of the two *Dscam2* mutants was weaker than the controls and in the opposite direction (Figure 3C). Finally, we maintained a constant spatial frequency and varied the speed of the bars to explore the temporal domain of the fly visual system sensitivity. Control flies responded well to all tested temporal frequencies. Neither of the two *Dscam2* mutants responded to lower temporal frequencies, but both elicited significant responses to faster moving bars. Consistent with the other tested parameters, the responses of both mutants were reversed compared to the controls (Figure 3D).

Although both *Dscam2* mutant strains showed a similar trend toward a negative visual response, the magnitudes of their responses were different, for most stimuli (Figures 3B–D). This was surprising given that these lines were backcrossed into the same genetic background as the control flies and the *Dscam2* mutations in the two strains were identical (Millard et al., 2007). These data raised the possibility that one of the lines contained a genetic modifier linked to the *Dscam2* mutation that was not removed during backcrossing. To address this, we tested a third line, again with an identical *Dscam2* mutation (*Dscam2*^*null*−3^), at different temporal frequencies. *Dscam2*^*null*−3^ turned against the direction of motion, like the two other mutants, but the magnitude of the response was more similar to *Dscam2*^*null*−1^ than *Dscam2*^*null*−2^ (Figure 3E). Although the responses of *Dscam2*^*null*−1^ and *Dscam2*^*null*−3^ were not significantly different from zero at lower temporal frequencies, the stimuli that induced the highest responses in control and *Dscam2*^*null*−2^ flies resulted in significant responses from these two lines as well (Figure 3E, Supplementary Table 1). Thus, all three mutants turn against the direction of motion even though the magnitudes of the responses are variable. We conclude that there is likely a genetic modifier independent of *Dscam2* in the *Dscam2*^*null*−2^ line that increases the magnitude of the visual response in the maze paradigm. Interestingly, this modifier appears to be assay-specific because it does not affect responses in the tethered-walking assay (see below).

### Specific motion parameters in a single fly assay elicit a negative optomotor response from *Dscam2* mutants

**Figure 4:**
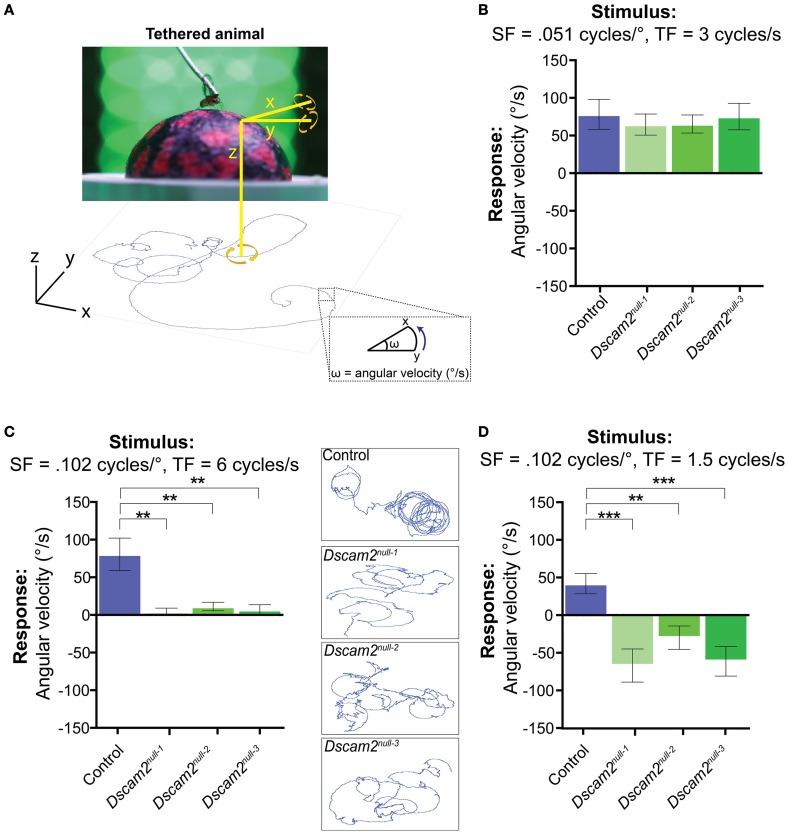
***Dscam2*** **mutant flies have conditional responses to motion in the tethered-walking assay. (A)** A tethered-walking assay to assess motion tracking in flies. This assay uses a tethered fly on an air-supported ball surrounded by LED panels displaying apparent motion. The movement of the ball is tracked by a camera and the software program FicTrac which translates rotation of the ball into a 2D fictive path. From this path the angular velocity can be determined and used as a measure of the response to the visual stimulus. **(B–D)** Visual responses of control (blue), *Dscam2*^*null*−1^ (light green), *Dscam2*^*null*−2^ (green) and *Dscam2*^*null*−3^ (dark green) to specific grating parameters in which the mutants have conditional responses; **(B)** Visual response (in angular velocity) to a standard grating at the indicated spatial and temporal frequencies (SF and TF, respectively). Control and mutant flies respond equally well. **(C)** Visual response to a grating with the indicated SF and TF where the control flies respond, but mutant flies do not. Insets represent the 2D fictive walking paths of single flies from each genotype. The traces of the *Dscam2* mutant paths illustrate their impaired rotational response. **(D)** Visual response to a grating with the same SF as in **(C)**, but a lower TF. Control flies turn toward the direction of motion whereas mutant flies turn against the motion. For all groups at least eight flies were run for every condition. Error bars indicate SEM. Significance indicated by asterisks in which ^**^p < 0.01, and ^***^p < 0.001.

The weak behavioral response of the *Dscam2* mutant flies in the maze paradigm raised the concern that these flies could be nearly motion blind. Although the maze paradigm is ideal for testing motion behavior in large populations of flies, the stimulus in the maze is complex. Light can be scattered by the glass and plastic from which the maze is constructed, flies have the ability to walk upside down in the maze channels, and social interactions with other flies can influence their decision at each choice point. Thus, the reduced magnitude and the inverted sign of the *Dscam2* mutant flies' visual response in the maze could involve many confounding factors. We turned to a tethered, single fly assay where we addressed these issues by carefully controlling what the fly sees. We first reasoned that we could increase the visual response of the mutants by putting them in an environment that was entirely focused on motion detection. In the tethered-walking assay a fly walks on an air-supported ball and is surrounded by LED panels where a moving grating of black and green stripes is presented. The movement of the air-supported ball is tracked by a camera and recorded by the software program FicTrac (Moore et al., 2014) (Figure 4A). FicTrac measures the rotation of the air-suspended ball and plots a 2D representation of the fly's path within the arena. The optomotor response is calculated based on the speed and direction of the ball in the presence of the moving grating and is expressed as an average angular velocity. A fly that is not walking will have no response whereas a fly that responds well to the stimulus will move the ball at a high angular velocity. As in the maze, following the motion stimulus results in a positive optomotor response.

We first tested the optomotor response of control flies using a moving grating stimulus with a contrast of 1, a spatial frequency of 0.051 cycles/°, and a temporal frequency of 3 Hz. This visual stimulus is similar to what was used in the maze, but the bars will appear narrower to the fly due to the increased distance between the fly and LED panels. In each experiment, flies were exposed to clockwise motion for 90 s, followed by a green screen for 60 s, and then counter-clockwise motion for 90 s (see Materials and Methods). A fly's optomotor response was calculated from the average angular velocity of the ball during the motion phases of the experiment. In control flies, the average angular velocity was variable and therefore multiple flies were tested with the same stimulus. Using the stimulus described above, control flies had a group average of 63 ± 14.0°/s (Mean ± SEM) (Figure 4B). This response was dependent on motion because a stationary grating did not elicit a directional response significantly different from zero (−5.6 ± 10.6°/s, *p* = 0.61, Figure S2). Flies followed clockwise and counter-clockwise motion with similar efficiencies, as expected. In contrast to the results from the maze, the three *Dscam2* mutants responded to the optomotor stimulus with positive angular velocities that were not significantly different from controls (Figure 4B). This demonstrated that the mutant flies are able to elicit an optomotor response similar to control flies under optimal conditions, and eliminated the possibility that they are motion blind.

As we did in the population visual response assay, we next challenged the capacity of the flies' visual system by presenting stimuli that are more difficult to detect. Control flies responded well to a wide range of spatial frequencies with angular velocities of 55–104°/s to each condition (Figure S3B). *Dscam2* mutants responded comparably to the different spatial frequencies with the exception of the highest spatial frequency, to which they did not respond (Figure 4C and Figure S3B). The limited range of the mutant's motion detection system is consistent with our data from the population assay. To explore this finding further, we used the spatial frequency where *Dscam2* mutant flies were not responding and tested a range of temporal frequencies. Control flies had some difficulty detecting the different temporal frequencies, responding to only two of the five conditions tested (Figure S3C). *Dscam2* mutant flies responded to one of the same conditions but interestingly, they responded in the opposite direction (Figure 4D and Figure S3C). Thus, we uncovered a specific visual parameter that caused the mutant flies to turn against the moving grating, consistent with our results from the population assay. We concluded from these experiments that the *Dscam2* mutant flies' detected motion as well as control flies under optimal conditions. However, they were unable to detect more challenging visual stimuli and they perceived motion in the opposite direction at a specific temporal frequency. These data make a strong argument that changes in visual system modularity can affect both the sensitivity of the motion detection circuitry and the perception of the visual stimulus.

### *Dscam2* mutant flies have defects in pattern-induced visual orientation

To investigate the visual perception of *Dscam2* mutant flies to other visual stimuli, we used a visual orientation stimulus that was independent of the optomotor response. Pattern-induced visual orientation behavior involves some of the neurons which are part of the motion circuitry but is thought to also involve pathways that are not related to motion detection (Tuthill et al., 2013). It has previously been observed that flies orient themselves in a stereotypic manner to different visual patterns. Orientation preferences can be monitored using a closed-loop system in which the fly is able to control the angular position of a bar in an arena. A dark bar on a light background causes the fly to place the bar either in its frontal quadrant (fixation) or in intermediate position between frontal and rear field of the fly (non-fixation), but preferentially in front. In contrast, a light bar on a dark background elicits the opposite response where the bar is placed preferentially behind the fly (anti-fixation) (Heisenberg and Wolf, 1979; Bülthoff, 1980; Wolf and Heisenberg, 1980). These orientation behaviors are dependent on the size of the bar (Maimon et al., 2008) and are somewhat assay-specific (Reiser and Dickinson, 2008).

In order to measure orientation behavior in *Dscam2* mutant flies, we converted our tethered-walking assay into a closed-loop system where the fly can turn the air-supported ball to control the position of the bar in the arena (Paulk et al., 2014). Every experiment consisted of multiple trials in which the bar was displaced 84° (at 280°/s) at random times by the computer. To ensure that the response of the bar displacement was independent of the optomotor response (in which the fly was following the moving grating), the bar was programmed to move in the opposite direction of the flies turning response.

**Figure 5:**
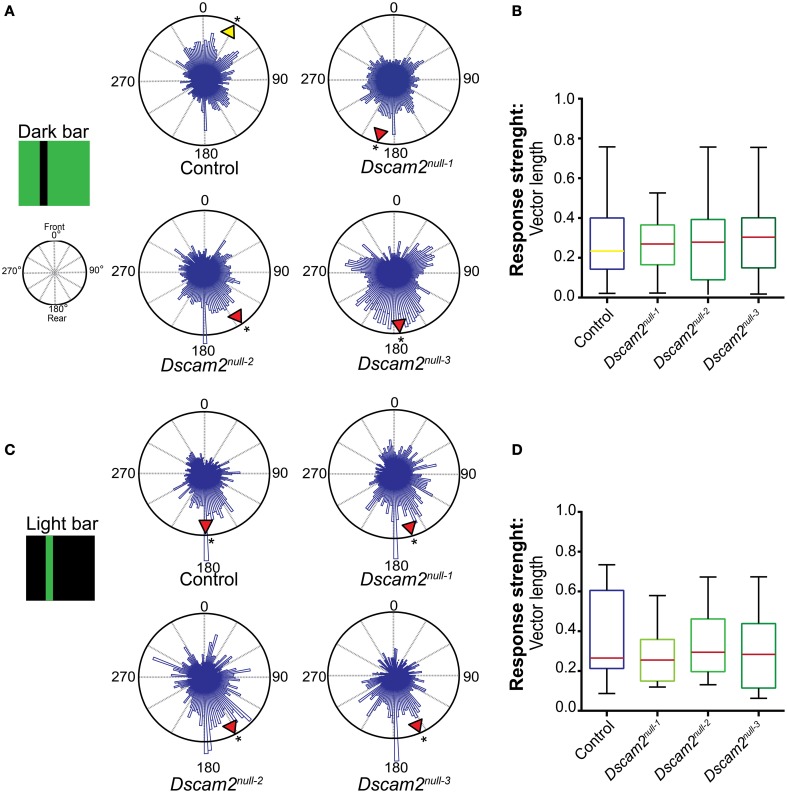
***Dscam2*** **mutant flies have defects in pattern-induced visual orientation**. Fixation Assay. Flies were presented with a bar in a closed-loop environment and their preference for the bar position was recorded. The histograms within the 360° circular plots represent a weighted value for each bar position derived from all of the flies from each genotype. For each fly an average direction was calculated, as well as a group mean direction which is graphically represented in the radial histogram as a yellow arrowhead for a heading toward the front of the arena or red arrowhead for a heading toward the back. A Rayleigh test confirmed the distributions were non-random, ^*^p < 0.05. **(A)** Control flies fixate and *Dscam2* mutants anti-fixate on a dark bar. **(B)** Control flies and *Dscam2* mutants demonstrate similar response strengths (indicated by median vector length) and variation for the fixation stimulus. The color of the median line corresponds with the color of the arrowhead (indicating direction) in **(A)**. **(C)** Control flies and *Dscam2* mutant flies both place a light bar toward the back of the arena. **(D)** Control flies and *Dscam2* mutants demonstrate similar response strengths (indicated by median vector length) and variation for the anti-fixation stimulus. As in **(B)**, the variation in strength of the response is visualized in boxplots with min to max whiskers. For all groups at least eight flies were run for every condition. A Kruskal-Wallis test indicated no significant difference between the vector lengths of the different fly strains.

We first tested the response of control flies to a dark bar on a light background. The data from multiple flies was plotted in a 360° circular graph (see Materials and Methods). In this plot the histogram length represents the weighting of each position. The mean direction (vector angle) and shape of the distribution (resultant vector length) was calculated for each fly. The group mean direction is graphically represented in the radial histogram with a yellow arrowhead if the direction was toward the front of the arena or with a red arrowhead if the direction was toward the back. Control flies elicited variable responses to a dark bar stimulus, but on average placed the bar toward the front of the arena (28°, 0° = front) (Figure 5A). In contrast to the control flies, all three *Dscam2* mutant strains elicited an anti-fixation response to this stimulus, placing the bar 145–195° from the front (Figure 5A). Rayleigh tests on the group mean direction showed a non-uniform distribution for both control and mutant flies (*p* < 0.01 and *p* < 0.05, respectively, Supplementary Table 2). Additional circular statistics are presented in Supplementary Tables 3 and 4. To compare the strengths and variation of the responses and we displayed these data in a box plot. Although the direction of the response was different between control and mutant flies, the strength and variability were not (Figure 5B). These data demonstrated that *Dscam2* mutant flies prefer to place a dark bar behind them, a response that is opposite to control flies.

We next tested the flies' response to a light bar on a dark background. This stimulus elicits anti-fixation behavior from wild-type flies in some behavioral paradigms (Heisenberg and Wolf, 1979; Bülthoff, 1980; Wolf and Heisenberg, 1980). Indeed, control flies demonstrated anti-fixation behavior with this stimulus by placing the bar 184° from the front (Figure 5C). Interestingly, mutant flies behaved similar to controls when presented with this stimulus, placing the bar 151–163° from the front (Figure 5C). Rayleigh tests on the group mean direction showed a non-uniform distribution for both control and mutant flies (*p* < 0.05, Supplementary Table 2). Additional circular statistics are presented in Supplementary Tables 3 and 4. The vector lengths and variability were similar in mutant and control flies (Figure 5D). Thus, the mutant flies perceive these stimuli in the same way. This suggests that defects in visual system processing are specific for particular behaviors in the mutant animals and are consistent with our results from the motion tracking assays.

## Discussion

In this study, we explored different visual behaviors in *Dscam2* mutant flies. We found that light and motion detection are impaired in these flies as would be expected for animals with defects in the organization and number of modules in the compound eye. Interestingly, *Dscam2* mutant flies elicit behavioral responses that are opposite to that of controls in three different assays. This behavioral phenotype is only observed under specific experimental conditions suggesting that Dscam2 is necessary for the wiring of a subset of circuits in the visual system that control the perception of visual stimuli.

### Dscam2-mediated modularity in the optic lobe is necessary for the correct optomotor response

The *Drosophila* optomotor response has been key for testing models of motion detection. The Hassenstein-Reichardt EMD model has been used to explain motion detection in *Drosophila* (Hassenstein and Reichardt, 1956) and has remained largely intact for over 50 years. The EMD model relies on the modularity of the visual system for motion detection. A key component of this model is that a moving object sequentially activates two sampling points. Due to a delay in activation of the first point, the signals detected by these two points become coincident when motion is in the preferred direction and this is translated into a speed and direction. Motion in the non-preferred direction does not elicit a coincident response from these same sampling points. Given that *Dscam2* mutants have fewer modules than wild-type flies, we expected that their visual acuity would be compromised. This is what we observed; these animals had attenuated phototaxis and motion tracking compared to controls.

What was unexpected was that *Dscam2* mutants respond to motion in an opposite manner compared to controls. Moving gratings of alternating dark and light bars have classically been used to elicit an optomotor response from flies. The response varies depending on the particular assays. In assays where the fly is tethered, individuals turn or walk in the direction of the motion, a response that is thought to stabilize their visual world (Götz, 1964, 1968). In other assays, flies respond to a moving grating by turning against the direction of motion (Lee et al., 2001; Zhu and Frye, 2009). What regulates this behavioral switch between following and moving against motion is not known. Our data suggest that Dscam2 plays a crucial role in the perception of these motion stimuli. In two different motion-tracking assays, *Dscam2* mutant flies moved against the direction of motion, in contrast to control flies. This response was conditional, suggesting that the circuitry that controls this behavior is not completely dysfunctional in the mutant. Rather, Dscam2 is necessary for the perception of specific visual cues. Presumably, these behavioral phenotypes are due to defects in visual system modularity that also compromise visual system acuity. In the *Dscam2* mutant, about 30% of the lamina cartridges are fused (Millard et al., 2010), reducing the number of sampling points from 750 to ~500. Each fused cartridge contains double the number of neurons found in a single cartridge and synapses form between photoreceptors from one cartridge and postsynaptic cells from the other (Millard et al., 2010). For motion detection, this could lead to three different scenarios: (1) a comparison between two single sampling points, (2) a comparison between a single and fused sampling point and (3) a comparison between two fused sampling points. We speculate that motion tracking may be reversed because sampling units are no longer receiving the correct temporal information and this results in an inversion in the sign of the response.

We considered the possibility that the direction of motion is misperceived due to aliasing. Aliasing is the misperception of the direction of motion due to physical constraints within the compound eye. When a grating has a spatial wavelength shorter than two times the interommatidial angle, aliasing occurs and the fly perceives motion in the opposite direction (Götz, 1964, 1965; Buchner, 1976). Although we cannot rule it out, we believe that aliasing is an unlikely explanation for the *Dscam2* mutant's inverted optomotor response, particularly in the maze. In the maze experiments, 25° was the minimum spatial wavelength attainable in this assay and a spatial wavelength of 9.6° would have been required to induce aliasing in wild-type flies. In *Dscam2* mutants that have 30% of their lamina cartridges fused, it is difficult to estimate what the “average” interommatidial distance because not every sampling point is affected. However, if for simplicity, if we assumed that every cartridge was fused then the interommatidial distance would double and a spatial wavelength of 19.2° would induce aliasing in the mutants. Since this is an overestimation of the interommatidial distance in the mutant and this spatial wavelength is less than what we could produce in the maze, we think that aliasing is a highly unlikely explanation. In the tethered-walking assay, there is less evidence against aliasing. The mutant flies responded negatively to a spatial wavelength of around 10°, which is close to the wild-type interommatidial angle. However, this stimulus did not induce aliasing in our control flies. This could be due to the asymmetrical shape of the LED arena, which results in different distances from the front and back LED panels to the fly (9.8 and 16.7°, respectively). It is therefore possible that the inversed behavior of the mutants on the ball is due to aliasing, but this would be inconsistent with the results from the maze.

### Pattern-induced visual orientation behavior

Apart from the changes in motion detection in *Dscam2* mutant flies, we also uncovered a change in pattern-induced visual orientation behavior. Visual orientation is crucial for insects; they use it for tracking and chasing prey as well as landing (Collett and Land, 1975). Using a cylindrical drum with black bars of different sizes, Wehner (1972) demonstrated that flies are attracted to dark objects and walk toward them. R1–6, R8 (Coombe, 1984), and L1–L2 (Tuthill et al., 2013) neurons have been implicated in this orientation behavior, called fixation. Fixation likely involves neurons that are not involved in the optomotor response as is suggested by the optomotor blind (*omb*^*H*31^) mutants. These flies lack a subset of lobula-plate giant fibers in the brain and they fixate, but do not respond to a moving grating (Heisenberg et al., 1978; Bausenwein et al., 1986), suggesting a separation of the optomotor and fixation circuitry. *Dscam2* mutant animals have wiring defects in R1–6, R8, and L1–L2 neurons. Interestingly, the mutants place a dark bar behind them, a response that is opposite to that of controls. In contrast to the fixation stimulus, a light bar on a dark background elicited an anti-fixation response similar to control flies. As with motion detection, Dscam2 appears to be necessary for the perception of specific visual cues. Although the cellular requirements for anti-fixation have not been as well defined as those for fixation, many of the same neurons are likely involved. Since fixation is affected but anti-fixation is normal, it could suggest that there are two different circuits downstream of the photoreceptor synapses, possibly in visual integration centers, that regulate these behaviors.

### Neural substrate of the inverted response

Inverted optomotor responses have been observed in other studies. One example is the zebrafish *belladonna (bel)* mutant. Some homozygous *bel* larvae display a reversed optomotor response when presented with a moving grating (Neuhauss et al., 1999). In these larvae a portion of the optic nerve fibers misroute in the optic chiasm, resulting in projection to the wrong hemisphere (Neuhauss et al., 1999). The chiasms of the *Dscam2* mutant are morphologically normal, so the *bel* mutant demonstrates the importance of correct wiring for the optomotor response, but it does not offer an explanation for our results.

Another study demonstrated an inversion of optomotor and object detection in the fly *Calliphora erythrocephala* and *D. melanogaster* using the GABA-antagonist picrotoxinin (Bulthoff and Bulthoff, 1987). The fly was placed on a rotatable ball in front of a monitor where moving gratings of light and dark bars were displayed. Wild-type flies showed a normal optomotor response to these moving gratings but turned against the direction of motion when injected with picrotoxinin. Fixation was also tested using a set-up similar to the one used in this study with the exception that the tethered flies were flying rather than walking. Untreated flies demonstrated fixation to a dark bar by placing it in front of them, however injection of picrotoxinin reversed their behavior to an anti-fixation response. The effect of picrotoxinin with an anti-fixation stimulus was not investigated. These studies suggest that inhibitory neurons play an important role in both optomotor and fixation responses and raise the possibility that Dscam2 plays a role in establishing the balance between inhibitory and excitatory inputs.

Interestingly, GABA signaling has also been linked to cognitive defects in a mouse model of Down syndrome. Ts65Dn mice are trisomic for two thirds of the human chromosome 21 homologs in the mouse, including DSCAM, and have cognitive defects that are consistent with Down syndrome. The Ts65Dn mice have increased inhibitory signaling in their brain (Kurt et al., 2000). Chronic treatment of these mice with a low doses of picrotoxin suppressed many of these cognitive phenotypes (Fernandez et al., 2007). Thus, this mouse model of disease where DSCAM is overexpressed has too much GABA signaling and our loss of function *Dscam2* mutants behave like flies with reduced inhibitory tone. This suggests that Dscam2 could play a role in establishing the appropriate excitatory/inhibitory ratio in the fly brain, but this remains to be tested.

In summary, previous studies demonstrated that Dscam2 plays a crucial role in establishing modularity in the optic lobe. In the absence of Dscam2, there are fewer modules and there is inappropriate cross talk between neighboring modules. Given these morphological phenotypes, the detriments in light and motion detection that we observed here were expected in the *Dscam2* mutants. What was unexpected was a role for Dscam2 in the perception of visual cues. To our knowledge, this is the first time that modularity has been linked to how visual stimuli are perceived by the brain.

### Conflict of interest statement

The authors declare that the research was conducted in the absence of any commercial or financial relationships that could be construed as a potential conflict of interest.
