# Supplementary material for: Dscam2 affects visual perception in Drosophila melanogaster
Source: Front Behav Neurosci. 2015 Jun 9;9:149. doi: 10.3389/fnbeh.2015.00149 (PMC4460526; doi:10.3389/fnbeh.2015.00149)
Supplement: Supplementary file 1 [file Image1.PDF]

Supplementary Material

Dscam2 affects visual perception in *Drosophila melanogaster*

Danny S. Bosch[1], Bruno van Swinderen[2], S. Sean Millard[1*]

[1]Millard laboratory, School of Biomedical Sciences, University of Queensland, St. Lucia, Queensland, Australia
[2]van Swinderen, Queensland Brain Institute, University of Queensland, St. Lucia, Queensland, Australia

*** Correspondence:** S. Sean Millard, Millard laboratory, School of Biomedical Sciences, University of Queensland, St. Lucia, Queensland, 4072, Australia
s.millard@uq.edu.au

Supplementary Figures

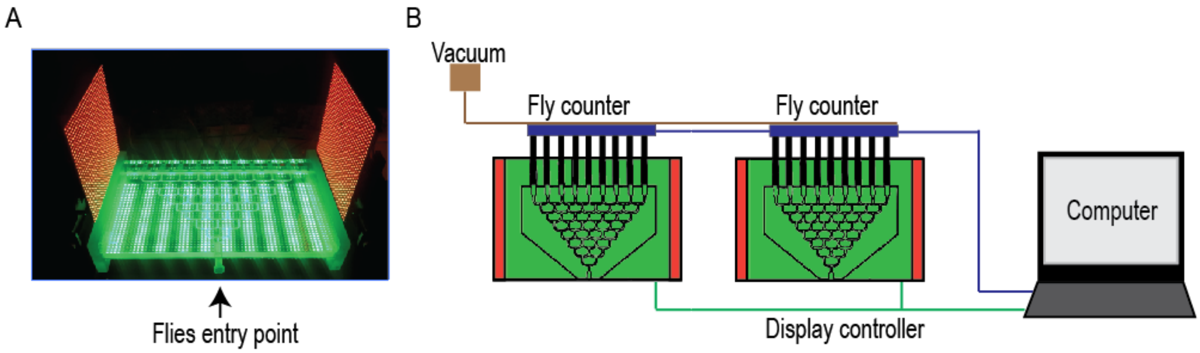

Figure S1. The maze visual response assay.

(A) A maze over a LED box where visual stimuli are displayed. The two red LED side panels provided a standard surrounding luminosity without color cues (flies are unable to see red). **(B)** A schematic of the complete set-up consisting of two LED boxes controlled by a computer. Fly counter automatically counts the flies as they enter the exit tubes. Flies are vacuumed out of the set-up after being counted.

Table S1. Mean visual responses and significance from zero for control and mutant flies to different visual stimuli in the maze visual response assay.

Mean visual responses of control, *Dscam2*$^{null\text{-}1}$, *Dscam2*$^{null\text{-}2}$, and *Dscam2*$^{null\text{-}3}$ to different contrast levels, spatial frequencies and temporal frequencies to supplement figure 2. Student's *t*-test against zero for normal distributed data and Mann-Whitney test against zero for non-normally distributed data. For all groups at least 8 mazes of approximately 30 flies each were run for every condition. *p* values < 0.05 are highlighted in green.

	Control		*Dscam2*$^{null\text{-}1}$		*Dscam2*$^{null\text{-}2}$		*Dscam2*$^{null\text{-}3}$	
	Mean response	Significance from 0	Mean response	Significance from 0	Mean response	Significance from 0	Mean response	Significance from 0
**Contrast curve (Figure 2B)**								
0.1	0.150	2.18E-01	0.116	3.74E-01	-0.030	8.99E-01	ND	ND
0.3	0.518	1.10E-04	-0.163	1.44E-01	-0.323	1.58E-01	ND	ND
0.5	0.595	1.73E-04	-0.221	5.16E-02	-0.143	3.57E-01	ND	ND
0.7	0.703	2.60E-03	-0.176	8.46E-02	-0.483	7.20E-03	ND	ND
1	0.642	4.32E-05	-0.161	1.95E-01	-0.536	8.20E-03	ND	ND
**Spatial Frequency Curve (cycles per degree, Figure 2C)**								
0.0095	1.017	1.12E-04	0.089	6.06E-01	-0.455	3.50E-03	ND	ND
0.0139	0.825	4.80E-04	-0.056	7.24E-01	-0.420	1.95E-02	ND	ND
0.0182	0.702	4.90E-11	-0.121	3.51E-01	-0.536	8.20E-03	ND	ND
0.027	0.702	1.70E-03	-0.114	4.64E-01	-0.275	6.52E-02	ND	ND
0.0314	0.660	3.50E-03	0.111	5.56E-01	-0.147	2.10E-01	ND	ND
0.0358	0.369	7.07E-02	0.062	5.89E-01	-0.082	6.56E-01	ND	ND
0.0402	0.061	6.15E-01	-0.059	6.39E-01	-0.071	6.57E-01	ND	ND
**Temporal Frequency curve (cycles per second, Figure 2D/E)**								
3	0.702	4.98E-11	-0.161	1.95E-01	-0.567	9.20E-03	-0.097	6.63E-01
4	0.778	4.40E-04	0.071	6.73E-01	-0.309	2.56E-02	ND	ND
6	0.269	4.80E-02	-0.265	1.10E-01	-0.563	2.00E-01	-0.299	1.78E-01
8	0.700	2.70E-07	-0.202	2.09E-01	-0.830	4.04E-05	-0.263	1.05E-01
9	0.449	1.05E-02	-0.309	3.86E-02	-0.863	7.56E-04	-0.484	6.70E-02
16	0.916	1.90E-06	-0.329	1.92E-02	-0.734	6.32E-06	-0.730	6.20E-03

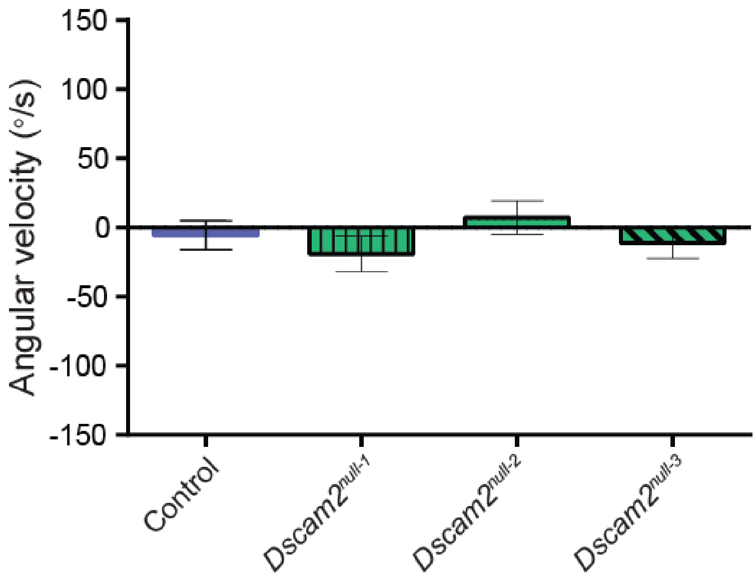

Figure S2. Control and mutant responses to a static grating in the tethered-walking assay.

Visual responses in the tethered-walking assay of control and *Dscam2* mutant flies to a static grating of green and black bars with a bar width of 19.6 ° (similar to the bar size used with a spatial frequency of 0.051 cycles/°). For all groups at least 6 flies were run. Error bars indicate SEM.

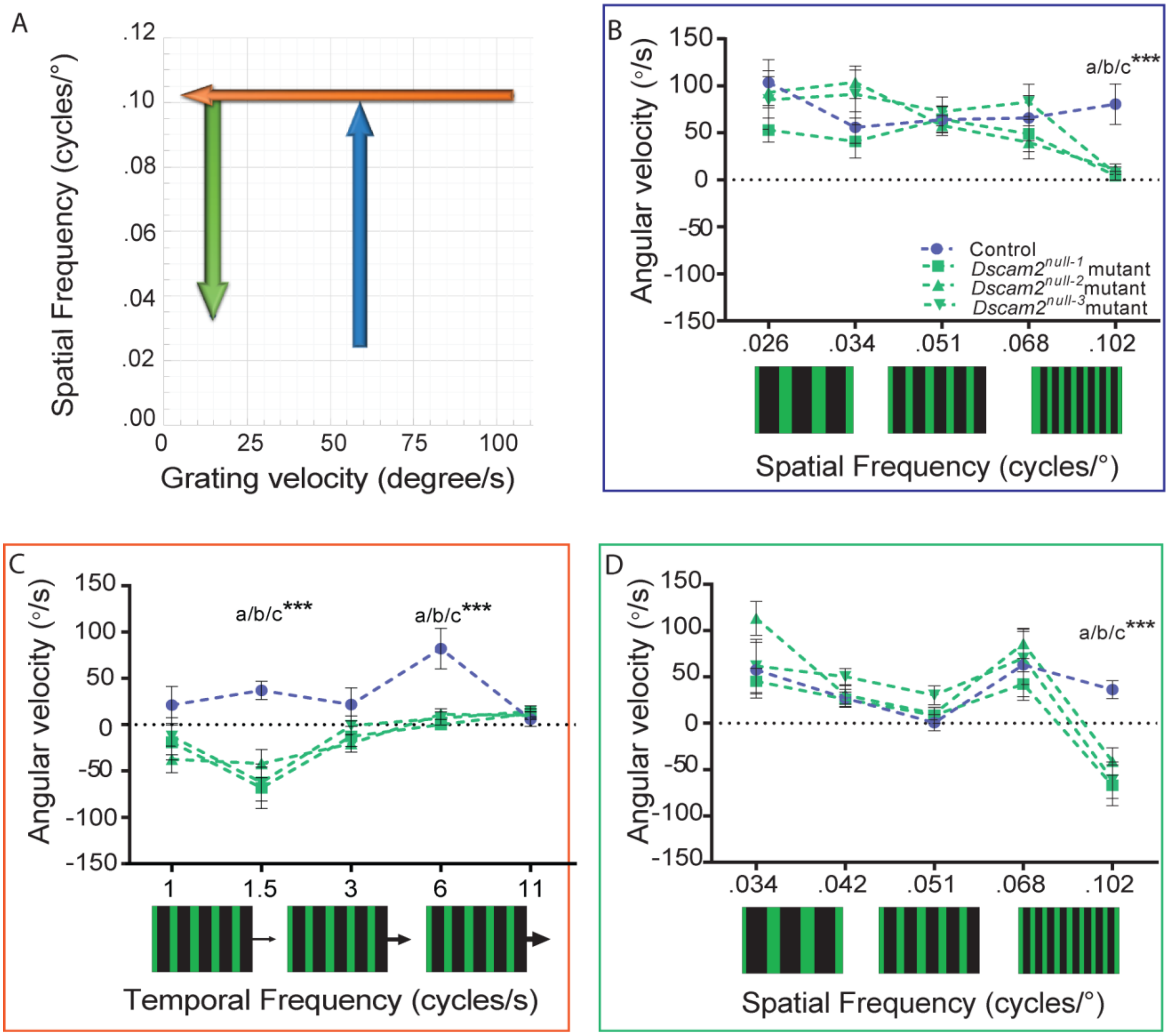

Figure S3. *Dscam2* mutant flies have conditional responses to motion in the tethered-walking assay.

(A) A schematic of the different spatial and temporal frequencies explored in this experiment, colors correspond to the outlines of the graphs**. (B-E)** Visual responses of control (blue), *Dscam2*$^{null\text{-}1}$ (green squares) and *Dscam2*$^{null\text{-}2}$ (green up-pointing triangles) and *Dscam2*$^{null\text{-}3}$ (green down-pointing triangles) flies to gratings with different spatial and temporal frequencies. **(B)** Visual response to different spatial frequencies at a constant grating velocity of 58.8 °/second; **(C)** various temporal frequencies at a constant spatial frequency of 0.051 cycles/° and **(D)** various spatial frequencies at a constant grating velocity of 14.7 °/second. Student's *t*-test performed for normal distributed data and Mann-Whitney test for non-normal distributed data. Significant differences indicated for **(a)** control versus *Dscam2*$^{null\text{-}1}$ mutant, (**b**) control versus *Dscam2*$^{null\text{-}2}$ and **(c)** control versus *Dscam2*$^{null\text{-}3}$ by asterisk in which *** $p < 0.001$. For all groups at least 8 flies were run for every condition. Error bars indicate SEM.

Table S2. Rayleigh test for non-uniformity of mean direction of individual genotypes.

Mean direction and vector length for each fly strain was calculated (7-12 displacements/fly) for each genotype. A Rayleigh test was performed on the individual means per genotype to test for uniformity of the circular data. p values < 0.05 are highlighted in green.

Stimulus	Fly strain	Mean (degrees)	Vector length	n	Rayleigh test
**Dark bar**	**Control**	28.22	0.444	30	0.002
	***Dscam2**$^{null-1}$	194.95	0.361	24	0.042
	***Dscam2**$^{null-2}$	144.79	0.558	23	0.000
	***Dscam2**$^{null-3}$	171.17	0.662	14	0.001
**Light bar**	**Control**	183.51	0.406	21	0.029
	***Dscam2**$^{null-1}$	163.12	0.497	13	0.037
	***Dscam2**$^{null-2}$	151.03	0.571	18	0.002
	***Dscam2**$^{null-3}$	158.76	0.609	14	0.004

Table S3. One-sample test for the mean angle.

Mean visual responses of control, *Dscam2*$^{null\text{-}1}$, *Dscam2*$^{null\text{-}2}$, and *Dscam2*$^{null\text{-}3}$ were compared to 0 ^{o} and 180 ^{o} using a one-sample test for the mean angle. This test is equivalent to a one-sample *t*-test with a specified mean direction. h=1 if the mean direction of the population is different from the specified mean direction.

Stimulus	Fly strain	Against 0 degrees	Against 180 degrees
**Dark bar**	**Control**	h=0	h=1
	Dscam2$^{null\text{-}1}$	h=1	h=0
	Dscam2$^{null\text{-}2}$	h=1	h=0
	Dscam2$^{null\text{-}3}$	h=1	h=0
**Light bar**	**Control**	h=1	h=0
	Dscam2$^{null\text{-}1}$	h=1	h=0
	Dscam2$^{null\text{-}2}$	h=1	h=0
	Dscam2$^{null\text{-}3}$	h=1	h=0

Table S4. Comparison of the population distribution by use of a Watson-Williams multiple-sample test for equal means.

Comparison of the population of mean directions of control, *Dscam2*$^{null-1,}$ *Dscam2*$^{null-2}$, and *Dscam2*$^{null-3}$ by use of parametric Watson-Williams multi-sample test. This tests for equal means and can be used as a one-way ANOVA test in which a $p < 0.05$ indicates that the populations have unequal means. p values < 0.05 are highlighted in green.

Stimulus		*p*-value
Dark bar	Control vs *Dscam2*$^{null-1}$	NA*
	Control vs *Dscam2*$^{null-2}$	1.2E-6
	Control vs *Dscam2*$^{null-3}$	4.7E-8
	Dscam2^{null-1} vs *Dscam2*$^{null-2}$	0.3689
	Dscam2^{null-1} vs *Dscam2*$^{null-3}$	0.3559
	Dscam2^{null-2} vs *Dscam2*$^{null-3}$	0.0403
Light bar	Control vs *Dscam2*$^{null-1}$	0.4803
	Control vs *Dscam2*$^{null-2}$	0.1885
	Control vs *Dscam2*$^{null-3}$	0.3038
	Dscam2^{null-1} vs *Dscam2*$^{null-2}$	0.6284
	Dscam2^{null-1} vs *Dscam2*$^{null-3}$	0.8047
	Dscam2^{null-2} vs *Dscam2*$^{null-3}$	0.7988

NA* Test not applicable. Average resultant vector length < 0.45
